# Comprehensive proteome profiling of cytochrome P450 isoforms in cancer models

**DOI:** 10.1186/s12014-025-09565-1

**Published:** 2025-10-31

**Authors:** Sadr ul Shaheed, Ahood A. Al-Eidan, Klaus Pors, Laurence Patterson, Chris W. Sutton

**Affiliations:** 1 Nuffield Department of Surgical Sciences and Biomedical Research Centre, University of Oxford, Oxford, UK; 2 NIHR Oxford Biomedical Research Centre, University of Oxford, Oxford, UK; 3 Department of Biology, College of Science, Imam Abdulrahman bin Faisal University, P. O. Box 1982, Dammam, 31441 Saudi Arabia; 4 Institute of Cancer Therapeutics, University of Bradford, Bradford, UK; 5 Nuffield Department of Surgical Sciences, John Radcliffe Hospital, Headley Way, Headington, Oxford, OX3 9BQ UK

**Keywords:** CYP450 enzymes, Colorectal cancer, Head and neck cancer, GEL-LC-MS, Biomarkers, Personalized therapy

## Abstract

**Background:**

Cytochrome P450 (CYP450) enzymes are essential for drug metabolism, xenobiotic detoxification, and procarcinogen activation, playing a pivotal role in both normal physiology and cancer biology. Their expression varies significantly across tissues and tumour types, reflecting the metabolic heterogeneity of cancers. Understanding these variations is critical for developing targeted therapies, optimizing drug efficacy, and minimizing toxicity. This study aimed to comprehensively profile CYP450 expression across colorectal cancer (CRC), head and neck squamous cell carcinoma (HNSCC), breast cancer, and hepatic cancer models using proteomic techniques.

**Methods:**

We analysed various cancer models (cell lines, xenografts, and patient tissue biopsies) using gel electrophoresis coupled with liquid chromatography-mass spectrometry (GEL-LC-MS). Equal amounts of protein were separated by gel electrophoresis, and the 45-65 kDa molecular weight range was analysed on the Orbitrap Fusion Mass Spectrometer.

**Results:**

Distinct CYP450 expression profiles were observed across cancer types. In CRC, CYP2W1 and CYP2S1 were highly expressed, while CYP1B1 and CYP2W1 were prominent in HNSCC, highlighting their potential as biomarkers and therapeutic targets. Breast cancer models predominantly expressed CYP2J2 and CYP2S1, whereas CYP3A and CYP2C subfamily members were enriched in hepatic cancer, underscoring their roles in xenobiotic metabolism and drug clearance.

**Conclusion:**

This study provides the first comprehensive semi-quantitative proteomic map of CYP450 isoforms across multiple cancer models. The findings reveal metabolic heterogeneity and identify clinically relevant targets, offering a foundation for future functional studies and personalized therapeutic strategies.

## Introduction

Cytochrome P450 (CYP450) enzymes are a superfamily of heme-containing monooxygenases that play a fundamental role in the metabolism of endogenous and exogenous compounds. These enzymes are involved in drug metabolism, xenobiotic detoxification, steroidogenesis, and the activation of procarcinogens, making them integral to both normal physiological processes and disease states, including cancer. The expression of CYP450 enzymes varies across tissues and tumour types, reflecting the metabolic heterogeneity of cancers and influencing drug efficacy, toxicity, and disease progression [1–3].

Understanding the differential expression of CYP450 enzymes in various cancers is essential for optimizing pharmacological interventions, predicting patient responses to treatment, and identifying novel therapeutic targets. Overexpression of certain CYP450 isoforms has been documented in various cancers, including breast, colorectal, lung, head and neck, gastric, oesophageal, hepatocellular, renal, childhood rhabdomyosarcoma, gall bladder carcinoma, and prostate cancers [4, 5]. Consequently, tumour-associated CYP450 isoforms, such as CYP1A1, CYP1B1, CYP2S1, and CYP2W1—have become a focus for the development of novel anticancer therapies [6]. Given the importance of CYP450 enzymes in cancer biology and treatment, comprehensive profiling of their expression in different tumour models is necessary to elucidate their functional significance and clinical implications [7].

Previous studies have employed various analytical techniques, including immunohistochemistry, Western blotting, and RNA sequencing, to investigate CYP450 expression. However, these methods have limitations in specificity and sensitivity, particularly in distinguishing between closely related CYP450 isoforms [8, 9]. Advances in proteomic technologies, such as liquid chromatography-mass spectrometry (LC-MS) [10], have provided a more precise and high-throughput approach for CYP450 profiling, allowing for the identification and quantification of these enzymes with greater accuracy.

In this study, we utilised gel electrophoresis coupled with liquid chromatography-mass spectrometry (GEL-LC-MS) to analyse the expression of CYP450 enzymes in CRC, HNSCC, breast cancer, and hepatic cancer models, including cell lines, xenografts, and patient tissue biopsies. The use of GEL-LC-MS offers several advantages, including improved protein separation, enhanced sensitivity, and the ability to identify CYP450 isoform-specific peptides. By focusing on the molecular weight range of 45–65 kDa, which encompasses most CYP450 enzymes, we aimed to generate a detailed expression profile and identify key isoforms involved in cancer metabolism.

## Materials and methods

### Clinical models

Cancer cell lines were sourced from the American Type Culture Collection (Manassas, VA). For the GEL-LC-MS analysis, the following cell lines were cultured under standard conditions to ensure optimal protein extraction. A panel of cancer cell lines representing multiple tumour types was included in this study: colorectal cancer (DLD1, HT-55, LS174T, COLO-205, HCC2998, C106, SW480-2W1, SW480-mock), head and neck squamous cell carcinoma (HNSCC) (A253, Detroit-562, FADU, DOK, OSC-19, SCC9), breast cancer (MCF-7), and hepatocellular carcinoma (HepG2). In addition, CHO-1A1 cells (CYP1A1-transfected Chinese hamster ovary cells) were included as a CYP450 overexpression model. Cells were maintained in the appropriate growth medium (RPMI-1640, DMEM, or DMEM/F-12), supplemented with 10% fetal bovine serum (FBS) and 1% penicillin–streptomycin, and incubated at 37 °C in a humidified atmosphere with 5% CO₂. Transfected cell lines were acquired as follows: CHO-1A1 cells were a kind gift from Thomas Freiburg (University of Dundee, UK), while SW480-2W1 and SW480-mock cells were kindly provided by Professor Magnus Ingelman-Sundberg (Karolinska Institute, Stockholm, Sweden). For harvesting, cells were detached by trypsin treatment, washed three times with sterile ice-cold PBS, and the resulting cell pellets (5 × 10⁶ cells) were stored at − 80 °C until further processing.

All animal procedures were conducted in accordance with UK National Cancer Research Institute guidelines for the Welfare of Animals. Immunodeficient Balb/C Nu/Nu mice aged 6 to 8 weeks were obtained from Harlan (Loughborough, UK). Human cancer cells were injected subcutaneously into these mice. Mice were sacrificed when tumours reached a volume of 300–500 mm³. Liver and xenograft tissues were extracted and stored at − 80 °C until required.

Human CRC tissue samples and head and neck cancer tissue samples were obtained from Ethical Tissue (ET), a Research Tissue Bank at the University of Bradford, approved by the University of Bradford’s Independent Scientific Advisory Committee (reference: application/13/051) and ethically approved by Leeds (East) Research Ethics Committee, reference 07/H1306/98 + 5.

### Sample preparation

**Fig. 1:**
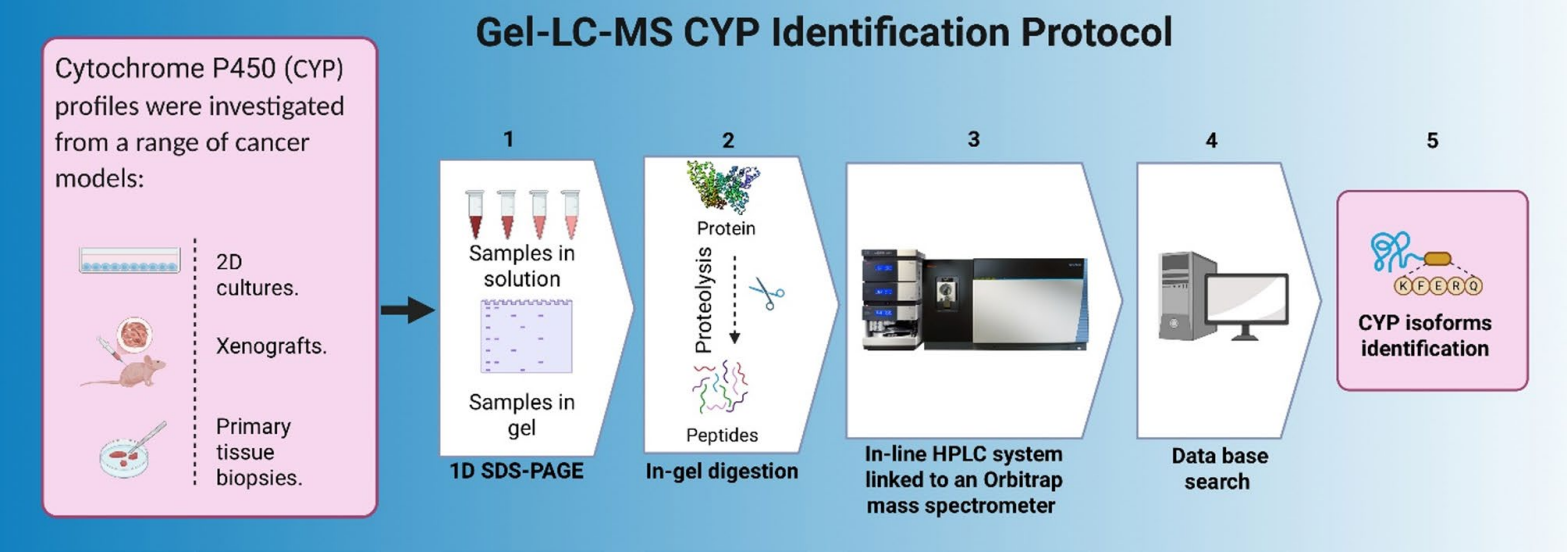
GEL-LC-MS workflow for the analysis of cell lines, tissue, and xenografts

Total protein was extracted from cell lines, xenografts, and human tissue biopsies and mouse livers using the cryo-pulverization method [11]. Mouse liver microsomes were prepared from the S9 fraction of liver extracts using the Beckman Optima TL100 Ultracentrifuge (Beckman Coulter, UK) as described previously [10]. After protein estimation of each sample using the Bradford assay (Sigma Aldrich, USA), 20 µg of each sample was reduced with Laemmli buffer at 90 °C for 5 min. Denatured lysates were resolved on 10% SDS-polyacrylamide gels using a Mini-PROTEAN 3 Cell system (Bio-Rad, USA). After electrophoresis, the gels were stained with Coomassie Blue reagent (PhastGel^®^ Blue R) for 1 h at room temperature and then destained with a solution containing 50% v/v methanol, 40% v/v water, and 10% v/v acetic acid. Bands corresponding to proteins in the molecular weight range of 45 kDa to 65 kDa were excised, diced into 1 mm³ pieces, and digested as described in previous studies [10]. Extracted peptides were lyophilized by vacuum centrifugation at 45 °C and stored at − 20 °C. The experimental workflow is outlined in Fig. 1, which illustrates the GEL-LC-MS approach used for the preparation and analysis of cancer cell lines, patient tissue biopsies, and xenograft models.

### LC-MS analysis

Lyophilized peptide fractions were reconstituted in 12 µl of loading mobile phase (2% acetonitrile (ACN)), 0.1% formic acid. (FA)), and 4 µl were loaded onto a C18 PepMap100 trapping cartridge (5 mm × 300 μm ID, 5 μm particle) (Thermo Scientific, UK) at a flow rate of 25 µl/minute on a Dionex Ultimate 3000 nanoHPLC (Thermo Scientific). After a 4-minute wash with the loading mobile phase, the trapping cartridge was connected to an Acclaim PepMap 100 column (25 cm × 75 μm ID, 2 μm particle) at a flow rate of 300 nl/minute with 5% mobile phase B (100% ACN, 0.1% FA) in mobile phase A (2% ACN, 0.1% FA). Several reverse phase elution gradients were used, extrapolated from a 100-minute gradient from 7 to 45% solvent B, followed by column wash and equilibration. Peptide elution was coupled to electrospray ionization (ESI) at 22 kV using a steel emitter and analysed with an Orbitrap Fusion mass spectrometer (Thermo Scientific, UK). MS1 data acquisition was conducted using Xcalibur 4.0 with Foundation 3.1 SP1 software (Thermo Scientific) between 350 and 1500 m/z at 120,000 mass resolution, automatic gain control (AGC) target 3 × 10^5^, RF lens ~ 60%, with a maximum injection time of 100 ms. MS/MS acquisitions were performed on the Ion-trap in top speed mode with a 3s cycle time, with a quadrupole isolation window of 1.2 m/z, dynamic exclusion (± 5 ppm) of 60 s, intensity threshold 5000, with all ions in charge states 2 + to 5 + sequentially fragmented by collision-induced dissociation (CID) with a normalized collision energy (NCE) of 35%. A maximum 50 ms ion injection time was allowed, AGC target set as 1 × 10^4^ and polysiloxane (C_2_H_6_SiO) at 445.12003 was used as a MS lock-mass. Each reconstituted in-gel digest sample was analysed in triplicate.

### GEL-LC-MS data analysis

Protein and peptide identification and quantification were performed using Proteome Discoverer 2.2, with Mascot software version 2.5 (Matrix Science, London, U.K.) against Swiss-Prot version 2022 containing 20,317 protein sequences (Human) and 17,135 (mouse). Proteins were identified by searching against reference human and mouse proteome databases, with trypsin as the proteolytic enzyme and allowing two missed cleavages. The mass tolerance for precursor and fragment ions was set to 5 ppm and 0.5 Da, respectively. Oxidation of methionine and deamidation of asparagine and glutamine were considered as variable modifications. Peptide spectrum match data were exported from Proteome Discoverer, including features such as protein name, number of peptides, unique peptides, number of PSMs, coverage, Mascot score, posterior error probability (PEP), charge states, and precursor intensity values. The list of protein identifications was manually assessed and filtered to include those with a protein Mascot score greater than the threshold value of 18 (equivalent to a significance of *p* ≤ 0.05), defined as Master Protein (≤ 1 unique peptide with peptide Mascot score greater than the threshold value of 18), and total PSMs ≥ 2. For visualization, protein abundance values were log₂-transformed prior to post-acquisition analysis and categorized into Low, Medium, and High expression groups by dividing the log₂ abundance distribution into tertiles (0–33%, 34–66%, 67–100%). All graphical and statistical analyses were conducted using Microsoft Excel 2016 and GraphPad PRISM software version 6.0 (GraphPad Software, San Diego, CA). The mass spectrometry proteomics data have been deposited to the ProteomeXchange Consortium via the PRIDE [12] partner repository with the dataset identifier PXD062999.

## Results

### Profiling of colorectal cancer models

**Fig. 2:**
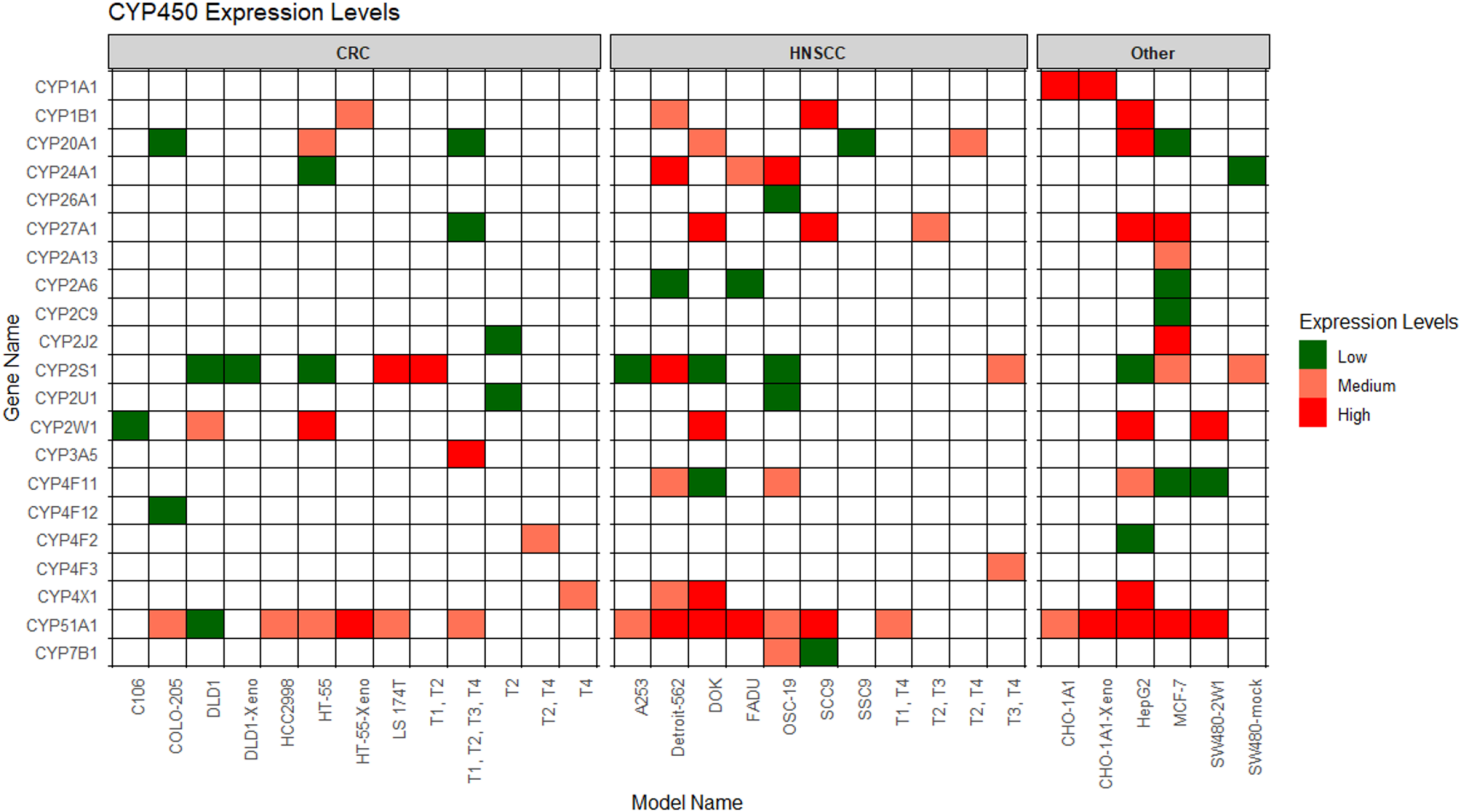
Heatmap of CYP450 Expression Levels Across Different Models and Sample Types. The heatmap displays the expression levels of CYP450 genes (rows) across different model types (columns), grouped by sample type. Gene IDs are sorted alphabetically (A to Z), and sample types are categorized into three groups: CRC, HNSCC, and Other (including Breast, Hepatoma, and Control). The color of each tile indicates protein expression level, with dark green = Low, coral = Medium, and red = High, defined by tertiles (0–33%, 34–66%, 67–100%) of the log₂-transformed abundance distribution

The analysis of colorectal cancer (CRC) models identified 16 CYP450 isoforms across various cell lines and tumour types (Table 1). CYP2W1 was particularly abundant in DLD1 cells, with 31% sequence coverage, 11 total peptides, and a Mascot score of 809. CYP1B1, CYP2J2, and CYP2S1 were detected in multiple models, with CYP2S1 exhibiting the highest coverage (15%) in HT-55 cells (Fig. 2; Table 1).

**Table 1:** Detection of CYP450 enzymes in human colorectal cancer models: CYP450 enzymes were identified in colorectal cancer cell lines, xenografts, and patient biopsies (stages T1–T4)

Gene	Accession	Protein	MW [kDa]	CRC model type	Coverage [%]	Total peptide	Unique peptides	PSMs	Mascot score	Abundances (log2)	Expression level
CYP1B1	Q16678	Cytochrome P450 1B1	60.8	HT-55-Xeno	7	3	3	14	115	23.97	Medium
CYP2J2	P51589	Cytochrome P450 2J2	55.8	T2	7	3	3	8	62	22.83	Low
CYP2S1	Q96SQ9	Cytochrome P450 2S1	55.8	DLD1	5	2	2	6	92	22.39	Low
				DLD1-Xeno	6	2	2	4	38	16.64	Low
				HT-55	15	4	3	12	188	20.94	Low
				LS 174T	13	5	5	14	215	26.59	High
				T1, T2	7	3	3	8	62	25.74	High
CYP2U1	Q7Z449	Cytochrome P450 2U1	53.8	T2	6	2	2	2	13	23.16	Medium
CYP2W1	Q8TAV3	Cytochrome P450 2W1	53.8	C106	13	5	5	8	77	19.10	Low
				DLD1	31	11	11	97	809	23.84	Medium
				HT-55	8	2	2	5	80	27.06	High
CYP3A5	P20815	Cytochrome P450 3A5	57.1	T1, T2, T3, T4	14	4	4	10	107	25.71	High
CYP4F2	P78329	Phylloquinone omega-hydroxylase CYP4F2	59.8	T2, T4	5	2	2	6	61	25.11	Medium
CYP4F12	Q9HCS2	Cytochrome P450 4F12	60.2	COLO-205	5	2	2	4	34	21.05	Low
CYP4 × 1	Q8N118	Cytochrome P450 4 × 1	58.8	T4	3	1	1	2	57	24.02	Medium
CYP20A1	Q6UW02	Cytochrome P450 20A1	52.4	COLO205	10	3	3	5	25	22.61	Low
				HT-55	10	4	2	14	119	23.88	Medium
				T1, T2, T3, T4	6	2	2	3	30	22.99	Medium
CYP24A1	Q07973	1,25-dihydroxyvitamin D(3) 24-hydroxylase, mitochondrial	58.8	HT-55	3	1	1	3	52	22.13	Low
CYP27A1	Q02318	Sterol 26-hydroxylase, mitochondrial	60.2	T1, T2, T3, T4	5	2	2	4	33	22.99	Medium
CYP51A1	Q16850	Lanosterol 14-alpha demethylase	56.8	COLO205	12	5	5	10	131	25.31	Medium
				DLD1	8	3	3	7	129	21.93	Low
				HCC2998	7	3	3	4	62	24.51	Medium
				HT-55	14	5	5	11	164	25.03	Medium
				HT-55-Xeno	28	14	9	5	302	27.62	High
				LS 174T	8	3	3	7	98	23.60	Medium
				T1, T2, T3, T4	6	2	2	4	85	24.24	Medium

The table summarizes gene symbol, UniProt accession, protein name, MW (kDa), CRC model, sequence coverage, peptide counts, PSMs, Mascot score, log₂ abundance, and qualitative expression (Low, Medium, High). Data are based on in-gel digestion of 45–65 kDa bands (*n* = 3 replicates)

The analysis of CRC patient tissue biopsies identified multiple CYP450 isoforms across different tumour stages, providing insight into their role in disease progression. CYP2J2 was detected in CRC stage pT2 with 7% sequence coverage and a Mascot score of 62, while CYP2S1 was found in early-stage CRC (T1, T2) with similar sequence coverage. The CYP3A5 and CYP51A1 enzymes were consistently expressed across all CRC stages (T1–T4) (Fig. 2; Table 1).

### Profiling of head and neck cancer models

The analysis of head and neck squamous cell carcinoma (HNSCC) models revealed a diverse expression of CYP450 isoforms across various cell lines and tumour stages (Table 2). CYP1B1 was among the most abundant enzymes, exhibiting 14% sequence coverage and a high Mascot score of 282 in SCC9 cells. CYP2S1 was detected across multiple models, including A253, Detroit-562, and OSC-19, while CYP2W1 was strongly expressed in DOK cells with 21% sequence coverage and a Mascot score of 172 (Fig. 2; Table 2).

**Table 2:** Detection of CYP450 enzymes in human head and neck squamous cell carcinoma models: CYP450 enzymes were identified in HNSCC cell lines, xenografts, and patient biopsies (stages T1–T4)

Gene	Accession	Protein	MW [kDa]	HNSCC model type	Coverage [%]	Total peptide	Unique peptides	PSMs	Mascot score	Average abundances (log2)	Expression level
CYP2A6	P11509	Cytochrome P450 2A6	56.5	Detroit-562	2	1	1	2	37	21.46	Low
				FADU	2	1	1	2	30	22.21	Low
CYP1B1	Q16678	Cytochrome P450 1B1	60.8	Detroit-562	7	3	3	7	91	24.99	Medium
				SCC9	14	5	3	14	282	26.04	High
CYP2S1	Q96SQ9	Cytochrome P450 2S1	55.8	A253	2	1	1	4	51	22.26	Low
				Detroit-562	6	3	2	8	104	26.40	High
				DOK	2	1	1	2	24	21.98	Low
				OSC-19	4	2	2	4	35	21.14	Low
				T3, T4	7	3	3	7	88	24.58	Medium
CYP2U1	Q7Z449	Cytochrome P450 2U1	61.9	OSC-19	4	1	1	2	26	22.55	Low
CYP2W1	Q8TAV3	Cytochrome P450 2W1	53.8	DOK	21	7	7	21	172	27.45	High
CYP4F11	Q9HBI6	Phylloquinone omega-hydroxylase CYP4F11	60.1	Detroit-562	5	2	2	4	103	25.44	Medium
				DOK	3	1	1	3	121	23.33	Medium
				OSC-19	8	3	3	5	107	24.14	Medium
CYP4F3	Q08477	Docosahexaenoic acid omega-hydroxylase CYP4F3	59.8	T3, T4	9	4	1	15	289	24.02	Medium
CYP4 × 1	Q8N118	Cytochrome P450 4 × 1	58.8	Detroit-562	3	1	1	2	35	25.26	Medium
				DOK	6	3	2	7	133	25.63	High
CYP7B1	O75881	25-hydroxycholesterol 7-alpha-hydroxylase	58.2	OSC-19	2	1	1	2	22	24.58	Medium
				SCC9	3	1	1	2	32	19.82	Low
CYP20A1	Q6UW02	Cytochrome P450 20A1	52.4	DOK	10	4	4	11	240	25.47	High
				SSC9	7	2	2	5	111	18.81	Low
				T2, T4	14	5	5	11	269	24.74	Medium
CYP24A1	Q07973	1,25-dihydroxyvitamin D(3) 24-hydroxylase, mitochondrial	58.8	Detroit-562	17	7	4	15	266	27.13	High
				FADU	5	2	2	3	67	23.46	Medium
				OSC-19	38	13	10	57	1126	29.71	High
CYP26A1	O43174	Cytochrome P450 26A1	56.2	OSC-19	3	1	1	2	18	20.41	Low
CYP27A1	Q02318	Sterol 26-hydroxylase, mitochondrial	60.2	DOK	35	12	12	41	527	28.72	High
				SCC9	12	4	4	10	69	26.35	High
				T2, T3	5	3	3	5	59	23.56	Medium
CYP51A1	Q16850	Lanosterol 14-alpha demethylase	56.8	A253	11	4	4	12	204	25.27	Medium
				Detroit-562	9	3	2	10	224	27.38	High
				DOK	26	8	8	21	470	26.51	High
				FADU	11	4	2	9	412	26.74	High
				OSC-19	13	4	4	9	108	25.10	Medium
				SCC9	17	6	3	15	213	26.40	High
				T1, T4	30	12	12	42	671	25.33	Medium

The table summarizes gene symbol, UniProt accession, protein name, MW (kDa), HNSCC model, sequence coverage, peptide counts, PSMs, Mascot score, log₂ abundance, and qualitative expression (Low, Medium, High). Data are based on in-gel digestion of 45–65 kDa bands (*n* = 3 replicates)

In HNSCC, CYP4F3 and CYP20A1 were extensively expressed in patient tissue biopsies from advanced stages (T2-T4), with CYP4F3 showing 9% coverage and a high Mascot score (289), and CYP20A1 exhibiting 14% coverage and a score of 269 (Table 2). CYP2S1 was significantly detected in T3 and T4 tumours, with a sequence coverage of 7% and a Mascot score of 88, while CYP20A1 was detected across pT2 and T4 tumours (Fig. 2; Table 2).

### Profiling of breast and hepatic cancer models

The analysis of breast (MCF-7) and liver (HepG2) cancer models revealed distinct CYP450 expression profiles, highlighting differential metabolic enzymes (Table 3). In HepG2 cells, CYP1B1 was prominently expressed, with 8% sequence coverage and a Mascot score of 212 and CYP2W1 was also detected in HepG2, with 17% sequence coverage and a Mascot score of 77 (Table 3). In MCF-7 breast cancer cells, multiple CYP450 isoforms were identified, including CYP2A6, CYP2A13, and CYP2J2, albeit at lower sequence coverages ranging from 1% to 7% (Fig. 2; Table 3).

**Table 3:** Detection of CYP450 enzymes in human breast Cancer, hepatocellular carcinoma and control cell lines

Gene	Accession	Protein	MW [kDa]	Cell lines/Xenografts	Coverage [%]	Total peptide	Unique peptides	PSMs	Mascot score	Abundances (log2)	Expression level
CYP1A1	P04798	Cytochrome P450 1A1	58.1	CHO-1A1	22	9	9	17	314	29.71	High
			58.1	CHO-1A1-Xeno	51	16	16	42	750	32.48	High
CYP1B1	Q16678	Cytochrome P450 1B1	60.8	HepG2	8	4	4	10	212	25.62	High
CYP2A6	P11509	Cytochrome P450 2A6	56.5	MCF-7	4	2	2	2	27	19.65	Low
CYP2A13	Q16696	Cytochrome P450 2A13	56.7	MCF-7	7	3	3	6	74	24.49	Medium
CYP2C9	P11712	Cytochrome P450 2C9	55.6	MCF-7	1	1	1	2	29	21.32	Low
CYP2J2	P51589	Cytochrome P450 2J2	57.6	MCF-7	4	2	2	4	55	25.94	High
CYP2S1	Q96SQ9	Cytochrome P450 2S1	55.8	HepG2	4	2	2	6	25	23.13	Medium
				MCF-7	3	1	1	2	66	25.21	Medium
				SW480-mock	5	2	2	5	29	24.43	Medium
CYP2W1	Q8TAV3	Cytochrome P450 2W1	53.8	HepG2	17	5	4	15	77	27.15	High
				SW480-2W1	31	10	7	54	668	31.38	High
CYP4F11	Q9HBI6	Phylloquinone omega-hydroxylase CYP4F11	60.1	HepG2	6	2	2	4	64	24.71	Medium
				MCF-7	3	1	1	2	60	23.23	Medium
				SW480-2W1	3	1	1	2	25	22.61	Low
CYP4F2	P78329	Phylloquinone omega-hydroxylase CYP4F2	59.8	HepG2	1	1	1	3	14	21.32	Low
CYP4 × 1	Q8N118	Cytochrome P450 4 × 1	58.8	HepG2	11	5	5	12	198	27.86	High
CYP20A1	Q6UW02	Cytochrome P450 20A1	52.4	HepG2	12	5	4	11	95	25.71	High
				MCF-7	8	3	3	7	45	22.25	Low
CYP24A1	Q07973	1,25-dihydroxyvitamin D(3) 24-hydroxylase, mitochondrial	58.8	SW480-mock	3	1	1	3	40	20.52	Low
CYP27A1	Q02318	Sterol 26-hydroxylase, mitochondrial	60.2	HepG2	22	9	8	30	410	27.78	High
				MCF-7	18	6	6	11	89	27.67	High
CYP51A1	Q16850	Lanosterol 14-alpha demethylase	56.8	CHO-1A1	5	2	2	4	93	24.14	Medium
				CHO-1A1-Xeno	13	5	5	9	189	28.54	High
				HepG2	17	6	5	15	274	26.79	High
				MCF-7	27	11	8	32	549	28.75	High
				SW480-2W1	18	6	5	14	278	26.47	High

CYP450 enzymes were detected in breast cancer, hepatocellular carcinoma, and control cell lines. The table summarizes gene symbol, UniProt accession, protein name, MW (kDa), cell line or xenograft model, sequence coverage (%), total and unique peptide counts, PSMs, Mascot score, log₂-transformed abundance, and qualitative expression level (Low, Medium, High). Data were obtained from in-gel digestion of 45–65 kDa protein bands (*n* = 3 replicates)

### Profiling of control CYP2W1 and CYP1A1 models

The analysis of CYP2W1 and CYP1A1 control cell lines provided further insights into their expression dynamics and functional significance (Table 3). CYP1A1 was highly abundant in CHO-1A1 transfected cells, with 22% sequence coverage and a Mascot score of 314, confirming strong detectability as would be expected (Table 4). CYP1A1 expression increased significantly in CHO-1A1 xenograft models, reaching 51% sequence coverage and a Mascot score of 750, suggesting enhanced enzyme stability or metabolic activity under in vivo conditions (Fig. 2; Table 3).

**Table 4:** Detection of CYP450 enzymes in mouse liver microsome

Gene	Accession	Protein	MW [kDa]	Coverage (%)	Total peptide	Unique peptides	PSMs	Mascot score	Abundances (log2)	Expression level
CYP1A1	P00184	Cytochrome P450 1A1	59.23	8	3	3	30	555	26.43	High
CYP1A2	P00186	Cytochrome P450 1A2	58.2	26	9	9	70	1019	27.26	High
CYP2A4	P15392	Cytochrome P450 2A4	56.782	45	15	4	209	1948	23.42	Medium
CYP2A5	P20852	Cytochrome P450 2A5	56.741	51	17	6	237	2425	26.08	High
CYP2A12	P56593	Cytochrome P450 2A12	56.179	48	16	15	169	2229	27.57	High
CYP2B9	P12790	Cytochrome P450 2B9	55.741	13	4	4	37	295	24.90	Medium
CYP2B10	P12791	Cytochrome P450 2B10	56.744	3	1	1	5	144	23.99	Medium
CYP2C29	Q64458	Cytochrome P450 2C29	55.716	34	11	5	146	1951	26.79	High
CYP2C37	P56654	Cytochrome P450 2C37	55.606	49	16	5	146	1660	25.72	High
CYP2C40	P56657	Cytochrome P450 2C40	55.763	22	9	9	111	989	28.45	High
CYP2C44	E9Q5K4	Cytochrome P450 2C44	56.422	24	8	8	45	781	25.28	Medium
CYP2C50	Q91 × 77	Cytochrome P450 2C50	55.8	45	15	4	221	2817	26.06	High
CYP2C54	Q6XVG2	Cytochrome P450 2C54	55.858	43	16	7	187	2200	26.50	High
CYP2C70	Q91W64	Cytochrome P450 2C70	56.02	22	8	8	32	354	25.83	High
CYP2D9	P11714	Cytochrome P450 2D9	56.95	14	5	2	33	552	23.57	Medium
CYP2D10	P24456	Cytochrome P450 2D10	57.233	60	21	12	413	4728	30.02	High
CYP2D11	P24457	Cytochrome P450 2D11	56.988	23	9	1	145	1382	23.69	Medium
CYP2D26	Q8CIM7	Cytochrome P450 2D26	56.976	37	13	11	166	1865	29.22	High
CYP2E1	Q05421	Cytochrome P450 2E1	56.805	34	11	11	97	843	28.25	High
CYP2F2	P33267	Cytochrome P450 2F2	55.949	48	15	15	209	2235	29.27	High
CYP2J5	O54749	Cytochrome P450 2J5	57.784	15	4	4	9	71	23.28	Medium
CYP3A11	Q64459	Cytochrome P450 3A11	57.855	45	12	6	1	1394	26.07	High
CYP3A13	Q64464	Cytochrome P450 3A13	57.492	18	6	6	32	443	24.41	Medium
CYP3A16	Q64481	Cytochrome P450 3A16	57.87	13	4	1	28	199	20.74	Low
CYP3A41	Q9JMA7	Cytochrome P450 3A41	57.987	24	8	3	83	881	24.68	Medium
CYP4A10	O88833	Cytochrome P450 4A10	58.33	5	2	2	4	54	21.76	Low
CYP4A14	O35728	Cytochrome P450 4A14	58.72	5	2	2	4	48	23.01	Medium
CYP4F3	Q99N16	Cytochrome P450 4 F	59.843	4	2	2	7	268	23.14	Medium
CYP4F14	Q9EP75	Leukotriene-B4 omega-hydroxylase 3	59.8	7	3	3	9	328	23.14	Medium
CYP17A1	P27786	Steroid 17-alpha-hydroxylase/17,20 lyase	57.638	18	6	6	17	94	25.32	Medium
CYP27A1	Q9DBG1	Sterol 26-hydroxylase, mitochondrial	60.72	50	15	15	223	1773	28.24	High
CYP51A1	Q8K0C4	Lanosterol 14-alpha demethylase	56.776	17	6	6	8	87	22.31	Low

CYP450 enzymes were detected in mouse liver microsomes. The table summarizes gene symbol, UniProt accession, protein name, MW (kDa), sequence coverage (%), total and unique peptide counts, PSMs, Mascot score, log₂-transformed abundance, and qualitative expression level (Low, Medium, High). Data were obtained from in-gel digestion of 45–65 kDa protein bands (*n* = 3 replicates)

CYP2W1 was prominently expressed in SW480-2W1 cells, with 31% sequence coverage and a high Mascot score of 668, reinforcing its robust detection. In contrast, its expression was minimal in SW480-mock cells, where only low levels of CYP2S1 (5% coverage) and CYP24A1 (3% coverage) were observed, while CYP4F11 was identified in SW480-2W1, at a lower abundance (3% coverage, Mascot score 25) (Fig. 2; Table 3).

### Profiling of mouse liver microsomes

Mouse liver CYP450 profiling was performed to allow comparison of the current strategy with the previous established platform employing GEL -MALDI-MS [10]. The GEL-LC-MS analysis of CYP450 proteins in mouse liver microsomes identified 32 CYP450 isoforms (Table 4), highlighting the diversity of CYP450 enzymes involved in hepatic metabolism. Among the identified proteins, CYP2D10 exhibited the highest sequence coverage (60%) and the highest Mascot score (4728), with 21 total peptides and 12 unique peptides. Other highly abundant CYP450 isoforms included CYP2A5 (51% coverage, Mascot score 2425) and CYP2C37 (49% coverage, Mascot score 1660), indicating their significant presence in the microsomal proteome (Table 4).

Several CYP2 family members, including CYP2A4, CYP2A12, CYP2C29, and CYP2C50, were also well represented with sequence coverages ranging from 34% to 48% and Mascot scores exceeding 1900 (Table 4). The CYP3A subfamily, critical for xenobiotic metabolism, was represented by CYP3A11, CYP3A13, and CYP3A16, with coverage values between 13% and 45%, and Mascot scores ranging from 199 to 1394. These isoforms are mouse homologs of human CYP3A4 and CYP3A5, which are the major hepatic CYP3A enzymes responsible for drug metabolism in humans. Given the high sequence similarity within the CYP3A subfamily (> 70–75% at the amino acid level), it is possible that antibodies raised against human CYP3A4 or CYP3A5 may cross-react with these murine orthologs. This underscores the importance of validating antibody specificity when interpreting immunodetection data across species or closely related isoforms. Lower-abundance CYPs, such as CYP4A10, CYP4A14, and CYP4F3, showed limited sequence coverage (4–5%) and low Mascot scores (< 300), suggesting either lower expression levels or reduced detectability in the microsomal fraction (Table 4). This may be due to their inherently low expression in the cancer models studied, limited solubility or stability in microsomal preparations, or fewer tryptic peptides suitable for MS detection.

## Discussion

LC-MS has emerged as a powerful analytical technique for profiling CYP450 enzymes, particularly due to its ability to resolve complex protein mixtures and provide high-resolution data on sequence coverage and peptide identification [9, 10, 13]. One of the key advantages of LC-MS is its unmatched precision in distinguishing highly homologous CYP450 isoforms, which pose challenges for traditional antibody-based detection methods. Consequently, some CYP450 isoforms have not previously been unequivocally detected in their protein form due to the difficulty in generating specific assays and monoclonal antibodies. However, GEL-LC-MS overcomes this challenge by combining the high-resolution separation of gel electrophoresis with the precise peptide mass detection of LC-MS, enabling the differentiation of closely related isoforms [10]. While DIA or other advanced LC-MS approaches offer higher throughput, they often struggle to detect low-abundance CYP450 isoforms due to signal suppression by abundant proteins. The Gel-LC-MS approach enriches the 45–65 kDa fraction, enhancing isoform-specific detection and enabling reliable semi-quantitative comparisons across samples.

The analysis of CRC, head and neck squamous cell carcinoma (HNSCC), and breast cancer models revealed distinct CYP450 expression profiles, highlighting tissue-specific metabolic adaptations that reflect the unique biochemical demands and microenvironmental conditions of each cancer type [14, 15]. In CRC models, the predominant expression of CYP2W1, CYP2S1, CYP1B1, and CYP2J2 suggests their potential functional involvement in tumour metabolism, xenobiotic processing and disease progression [16–19]. CYP2W1, in particular, has been previously associated with CRC and is known for its ability to metabolize pro-carcinogens into active compounds [16, 19]. The presence of CYP3A5 across all CRC patient biopsy stages T1 to T4, suggests its role in xenobiotic metabolism, possibly influencing drug response in CRC patients [20].

In head and neck squamous cell carcinoma (HNSCC) models, CYP1B1 was among the most abundant enzyme in SCC9 cells. CYP1B1 is known to metabolize a wide range of xenobiotics and is often overexpressed in cancer, making it a potential biomarker for HNSCC and a target for selective inhibitors [21–23]. The presence of CYP2W1 in HNSCC further reinforces its potential functional role in cancerous cells, expanding its relevance beyond CRC [23], while CYP2S1 has been implicated in inflammatory responses [24], potentially contributing to the aggressive nature of HNSCC tumours. Breast cancer models, particularly MCF-7 cells, exhibited a different CYP450 profile, with CYP2J2, CYP2S1, CYP2A6 and CYP2A13 being the most prominent isoforms. CYP2J2 has been implicated in the metabolism of chemotherapeutic agents, raising the possibility that its expression could influence drug resistance and treatment response [25]. Differential expression of CYP450 isoforms across cancer types has potential clinical implications, as these enzymes modulate drug metabolism, efficacy and toxicity. For example, elevated CYP2W1 in CRC and HNSCC could inform prodrug activation strategies, while CYP1B1 overexpression in HNSCC may guide selective inhibitor development. Linking these expression patterns to potential drug metabolism enhances the translational relevance of our dataset and may inform personalized therapeutic strategies.

The study also highlighted the influence of xenograft and tissue microenvironments on CYP450 expression. For example, CYP1A1 expression increased significantly in CHO-1A1 xenograft models, reaching 51% sequence coverage compared to 22% coverage in transfected cells. This suggests that the in vivo tumour microenvironment may enhance CYP1A1 stability or metabolic activity, possibly due to stromal interactions or extracellular matrix remodelling [26]. Similarly, CYP4F3 and CYP20A1 were prominently expressed in advanced-stage HNSCC patient biopsies, indicating that the tumour microenvironment may upregulate these isoforms to support cancer progression. These findings underscore the importance of considering the tissue context when studying CYP450 expression and designing therapeutic interventions.

GEL-LC-MS provides a highly sensitive and effective approach for enriching specific protein subgroups that are often undetectable in conventional total proteomic analyses. Notably, a search of the PRIDE database (https://www.ebi.ac.uk/pride/) did not reveal any datasets reporting the detection of CYP2W1 and CYP2S1 in colon or head and neck cancer models, underscoring the novelty of our findings. To further contextualize our results, we compared our GEL-LC-MS dataset with publicly available ProteomicsDB data (https://www.proteomicsdb.org/). This comparison identified 17 CYP isoforms consistently detected across seven common models (HepG2, SCC9, DOK, HT55, MCF-7, DLD1, HCC2998). Importantly, we detected CYP isoforms with established roles in drug metabolism; CYP2W1, CYP2J2, and CYP1A1, expressed uniquely in additional cancer cell lines (A253, C106, COLO-205, Detroit-562, FADU, LS174T, OSC-19, SSC9), transfected lines (CHO-1A1, SW480-2W1, SW480-mock), and xenografts (DLD-1-Xeno, HT55-Xeno, CHO-1A1-Xeno), which have not been previously reported. These findings demonstrate the ability of GEL-LC-MS to uncover low-abundance, drug-metabolizing CYP isoforms that may be missed in standard shotgun proteomics. However, a limitation of this approach is reduced accuracy in absolute protein quantification, primarily due to variability in trypsin digestion and peptide extraction from gels. Overall, our results highlight the unique strength of GEL-LC-MS for comprehensive, isoform-specific CYP450 profiling, providing novel insights into cancer-associated metabolic pathways and potential drug metabolism targets.

Log₂-transformed abundances and categorical expression levels (Low, Medium, High) enabled semi-quantitative comparisons of CYP450 expression across cancer models. Differential expression patterns suggest tissue-specific metabolic adaptations and have potential implications for drug metabolism. For example, CYP2W1, highly expressed in CRC and HNSCC models, represents an attractive target for prodrug activation strategies [16, 27], whereas CYP1B1 overexpression in HNSCC may guide selective inhibitor development. Similarly, CYP2J2 and CYP2S1 expression in breast cancer models could influence drug metabolism and treatment response. These findings highlight how isoform-specific CYP450 profiling can inform personalized therapeutic strategies. Microsomal enrichment and GEL-LC-MS provided sensitive detection of low-abundance isoforms, while comparison with public datasets (PRIDE, ProteomicsDB) confirmed the novelty of key drug-metabolizing CYPs uniquely detected in our models.

Microsomal isolation is a critical step in achieving in-depth CYP450 profiling [28–30]. Microsomes, vesicles derived from the endoplasmic reticulum, are enriched with CYP450 enzymes and other drug-metabolizing enzymes. Isolating microsomes allows researchers to focus on the functional fraction of the proteome, thereby enhancing the detection of low-abundance CYP450 isoforms. For instance, this study identified low-abundance CYPs such as CYP4A10 and CYP4A14 in mouse liver microsomes, demonstrating the utility of microsomal enrichment in overcoming detection challenges associated with whole-cell lysates. CYP450 profiles generated by GEL-LC-MS compared favourably with the previously established GEL-MALDI-MS strategy [10], with 22 CYP450 isoforms being common between the two approaches. Notably, CYP2C39, CYP3A25, and CYP8B1 were detected exclusively by GEL-LC-MS, while CYP17A1, CYP1A1, CYP2B10, CYP2C44, CYP2D11, CYP3A16, CYP4A10, CYP4F3, and CYP51A1 were only observed with GEL-MALDI-MS. These differences underscore the complementary nature of these techniques and their potential for comprehensive CYP profiling.

This study offers valuable insights into CYP450 expression across cancer models but has limitations. While GEL-LC-MS is effective for detecting specific isoforms, its sensitivity remains constrained by the dynamic range of protein expression, potentially leading to the omission of very low-abundance CYP450 enzymes. Targeted proteomic approaches, such as parallel reaction monitoring (PRM) or multiple reaction monitoring (MRM), could further enhance sensitivity and quantification accuracy. Additionally, this study focused on protein expression without assessing enzymatic activity, which is critical for functional relevance. The influence of post-translational modifications (PTMs), as well as genetic and epigenetic factors affecting CYP450 regulation, were not explored. Future studies incorporating activity assays, PTM analysis, and transcriptomic integration will provide a more comprehensive understanding of CYP450 function in cancer progression and drug metabolism.

## Conclusion

This study demonstrates the effectiveness of GEL-LC-MS for profiling CYP450 enzymes across diverse cancer models and mouse liver microsomes, enabling detection of low-abundance isoforms often missed by conventional approaches. Distinct CYP450 expression patterns reveal metabolic heterogeneity and identify potential therapeutic targets. Differential CYP450 expression can influence drug efficacy, toxicity, pharmacokinetics, and resistance, underscoring the value of these findings for personalized treatment strategies. Xenograft and tissue microenvironments further modulate CYP450 expression, highlighting the importance of biological context. Future studies should focus on functional validation, measurement of enzymatic activity, targeted proteomics (PRM/MRM), and integration of transcriptomic and post-translational modification analyses to enhance the translational relevance of CYP450 profiling.

## Data Availability

The mass spectrometry proteomics data generated in this study have been deposited to the ProteomeXchange Consortium via the PRIDE partner repository [12] with the dataset identifier PXD062999.

## References

[1] Pelkonen O, Turpeinen M, Hakkola J, Honkakoski P, Hukkanen J, Raunio H. Inhibition and induction of human cytochrome P450 enzymes: current status. Arch Toxicol. 2008;82:667–715. 18618097 10.1007/s00204-008-0332-8

[2] Ghosh C, Gonzalez-Martinez J, Hossain M, Cucullo L, Fazio V, Janigro D, Marchi N. Pattern of P450 expression at the human blood–brain barrier: roles of epileptic condition and laminar flow. Epilepsia. 2010;51(8):1408–17. 20074231 10.1111/j.1528-1167.2009.02428.x PMC3386640

[3] Renaud HJ, Cui JY, Khan M, Klaassen CD. Tissue distribution and gender-divergent expression of 78 cytochrome P450 mRNAs in mice. Toxicol Sci. 2011;124(2):261–77. 21920951 10.1093/toxsci/kfr240 PMC3216415

[4] Mittal B, Tulsyan S, Kumar S, Mittal RD, Agarwal G. Cytochrome P450 in cancer susceptibility and treatment. Adv Clin Chem. 2015;71:77–139. 26411412 10.1016/bs.acc.2015.06.003

[5] Zarrabi A, Perrin D, Kavoosi M, Sommer M, Sezen S, Mehrbod P, Bhushan B, Machaj F, Rosik J, Kawalec P. Rhabdomyosarcoma: current therapy, challenges, and future approaches to treatment strategies. Cancers. 2023;15(21):5269. 37958442 10.3390/cancers15215269 PMC10650215

[6] Sneha S, Baker SC, Green A, Storr S, Aiyappa R, Martin S, Pors K. Intratumoural cytochrome P450 expression in breast cancer: impact on standard of care treatment and new efforts to develop tumour-selective therapies. Biomedicines. 2021;9(3):290. 33809117 10.3390/biomedicines9030290 PMC7998590

[7] Norouzi-Barough L, Sarookhani MR, Sharifi M, Moghbelinejad S, Jangjoo S, Salehi R. Molecular mechanisms of drug resistance in ovarian cancer. J Cell Physiol. 2018;233(6):4546–62. 29152737 10.1002/jcp.26289

[8] Shrivas K, Mindaye ST, Getie-Kebtie M, Alterman MA. Mass spectrometry-based proteomic analysis of human liver cytochrome (s) P450. Toxicol Appl Pharmcol. 2013;267(1):125–36. 10.1016/j.taap.2012.12.008 23274569

[9] Williamson BL, Purkayastha S, Hunter CL, Nuwaysir L, Hill J, Easterwood L, Hill J. Quantitative protein determination for CYP induction via LC-MS/MS. Proteomics. 2011;11(1):33–41. 21182192 10.1002/pmic.201000456

[10] Sutton CW, Sutherland M, Shnyder S, Patterson LH. Improved Preparation and detection of cytochrome P450 isoforms using MS methods. Proteomics. 2010;10(2):327–31. 19902426 10.1002/pmic.200900489

[11] Shaheed S-u, Rustogi N, Scally A, Wilson J, Thygesen H, Loizidou MA, Hadjisavvas A, Hanby A, Speirs V, Loadman P. Identification of stage-specific breast markers using quantitative proteomics. J Proteome Res. 2013;12(12):5696–708. 24106833 10.1021/pr400662k

[12] Perez-Riverol Y, Bandla C, Kundu DJ, Kamatchinathan S, Bai J, Hewapathirana S, John NS, Prakash A, Walzer M, Wang S. The PRIDE database at 20 years: 2025 update. Nucleic Acids Res. 2025;53(D1):D543–53. 39494541 10.1093/nar/gkae1011 PMC11701690

[13] Liu X, Hu L, Ge G, Yang B, Ning J, Sun S, Yang L, Pors K, Gu J. Quantitative analysis of cytochrome P450 isoforms in human liver microsomes by the combination of proteomics and chemical probe-based assay. Proteomics. 2014;14(16):1943–51. 24920405 10.1002/pmic.201400025

[14] Kumarakulasingham M, Rooney PH, Dundas SR, Telfer C, Melvin WT, Curran S, Murray GI. Cytochrome p450 profile of colorectal cancer: identification of markers of prognosis. Clin Cancer Res. 2005;11(10):3758–65. 15897573 10.1158/1078-0432.CCR-04-1848

[15] Greer ML, Richman PI, Barber PR, Wilson GD, Murray GI, Patterson LH, Everett SA. Cytochrome P450 1B1 (CYP1B1) is expressed during the malignant progression of head and neck squamous cell carcinoma (HNSCC). Cancer Res. 2004;64(7Supplement):854–854.

[16] Travica S, Pors K, Loadman PM, Shnyder SD, Johansson I, Alandas MN, Sheldrake HM, Mkrtchian S, Patterson LH, Ingelman-Sundberg M. Colon Cancer–Specific cytochrome P450 2W1 converts duocarmycin analogues into potent tumor cytotoxins. Clin Cancer Res. 2013;19(11):2952–61. 23589180 10.1158/1078-0432.CCR-13-0238

[17] Alzahrani AM, Rajendran P. The multifarious link between cytochrome P450s and cancer. Oxidative Med Cell Longev. 2020;2020(1):3028387. 10.1155/2020/3028387 PMC6964729 31998435

[18] Aiyappa-Maudsley R, Storr SJ, Rakha EA, Green AR, Ellis IO, Martin SG. CYP2S1 and CYP2W1 expression is associated with patient survival in breast cancer. J Pathology: Clin Res. 2022;8(6):550–66. 10.1002/cjp2.291 PMC9535097 35902379

[19] Singh RD, Avadhesh A, Sharma G, Dholariya S, Shah RB, Goyal B, Gupta SC. Potential of cytochrome P450, a family of xenobiotic metabolizing enzymes, in cancer therapy. Antioxid Redox Signal. 2023;38(10–12):853–76. 36242099 10.1089/ars.2022.0116

[20] Buck E, Sprick M, Gaida MM, Grüllich C, Weber TF, Herpel E, Bruckner T, Koschny R. Tumor response to Irinotecan is associated with CYP3A5 expression in colorectal cancer. Oncol Lett. 2019;17(4):3890–8. 30881507 10.3892/ol.2019.10043 PMC6403523

[21] Shatalova EG, Klein-Szanto AJ, Devarajan K, Cukierman E, Clapper ML. Estrogen and cytochrome P450 1B1 contribute to both early-and late-stage head and neck carcinogenesis. Cancer Prev Res. 2011;4(1):107–15. 10.1158/1940-6207.CAPR-10-0133 PMC3043603 21205741

[22] Morvan VL, Richard É, Cadars M, Fessart D, Broca-Brisson L, Auzanneau C, Pasquies A, Modesto A, Lusque A, Mathoulin-Pélissier S. Cytochrome P450 1B1 polymorphism drives cancer cell stemness and patient outcome in head-and-neck carcinoma. Br J Cancer. 2020;123(5):772–84. 32565541 10.1038/s41416-020-0932-5 PMC7462978

[23] Presa D, Khurram SA, Zubir AZ, Smarakan S, Cooper PA, Morais GR, Sadiq M, Sutherland M, Loadman PM, McCaul J. Cytochrome P450 isoforms 1A1, 1B1 AND 2W1 as targets for therapeutic intervention in head and neck cancer. Sci Rep. 2021;11(1):18930. 34556703 10.1038/s41598-021-98217-z PMC8460628

[24] Bui P, Imaizumi S, Beedanagari SR, Reddy ST, Hankinson O. Human CYP2S1 metabolizes cyclooxygenase-and lipoxygenase-derived eicosanoids. Drug Metab Dispos. 2011;39(2):180–90. 21068195 10.1124/dmd.110.035121 PMC3033693

[25] Narjoz C, Favre A, McMullen J, Kiehl P, Montemurro M, Figg WD, Beaune P, de Waziers I, Rochat B. Important role of CYP2J2 in protein kinase inhibitor degradation: A possible role in intratumor drug disposition and resistance. PLoS ONE. 2014;9(5):e95532. 24819355 10.1371/journal.pone.0095532 PMC4018390

[26] Stevison F, Kosaka M, Kenny JR, Wong S, Hogarth C, Amory JK, Isoherranen N. Does in vitro cytochrome P450 downregulation translate to in vivo drug-drug interactions? Preclinical and clinical studies with 13‐cis‐retinoic acid. Clin Transl Sci. 2019;12(4):350–60. 30681285 10.1111/cts.12616 PMC6617839

[27] Sheldrake HM, Travica S, Johansson I, Loadman PM, Sutherland M, Elsalem L, Illingworth N, Cresswell AJ, Reuillon T, Shnyder SD. Re-engineering of the duocarmycin structural architecture enables bioprecursor development targeting CYP1A1 and CYP2W1 for biological activity. J Med Chem. 2013;56(15):6273–7. 23844629 10.1021/jm4000209

[28] Ravindranath V, Anandatheerthavarada HK. Preparation of brain microsomes with cytochrome P450 activity using calcium aggregation method. Anal Biochem. 1990;187(2):310–3. 2382832 10.1016/0003-2697(90)90461-h

[29] DuBois BN, Amirrad F, Mehvar R. A comparison of calcium aggregation and ultracentrifugation methods for the Preparation of rat brain microsomes for drug metabolism studies. Pharmacology. 2021;106(11–12):687–92. 34662883 10.1159/000519667

[30] Rasmussen MK, Ekstrand B, Zamaratskaia G. Comparison of cytochrome P450 concentrations and metabolic activities in Porcine hepatic microsomes prepared with two different methods. Toxicol in Vitro. 2011;25(1):343–6. 20940039 10.1016/j.tiv.2010.10.007

